# Food Xanthan Polysaccharide Sulfation Process with Sulfamic Acid

**DOI:** 10.3390/foods10112571

**Published:** 2021-10-25

**Authors:** Aleksandr S. Kazachenko, Natalya Yu. Vasilieva, Valentina S. Borovkova, Olga Yu. Fetisova, Noureddine Issaoui, Yuriy N. Malyar, Evgeniy V. Elsuf’ev, Anton A. Karacharov, Andrey M. Skripnikov, Angelina V. Miroshnikova, Anna S. Kazachenko, Dmitry V. Zimonin, Vladislav A. Ionin

**Affiliations:** 1 Institute of Non-Ferrous Metals and Materials Science, Siberian Federal University, pr. Svobodny 79, 660041 Krasnoyarsk, Russia; vasilyeva.nata@mail.ru (N.Y.V.); bing0015@mail.ru (V.S.B.); yumalyar@gmail.com (Y.N.M.); and-skripnikov@yandex.ru (A.M.S.); miroshnikova35@gmail.com (A.V.M.); kaalla@list.ru (A.S.K.); zimonind89@mail.ru (D.V.Z.); sl79490@yandex.ru (V.A.I.); 2 FRC “Krasnoyarsk Science Center”, Institute of Chemistry and Chemical Technology, Siberian Branch, Russian Academy of Sciences, Akademgorodok 50/24, 660036 Krasnoyarsk, Russia; fou1978@mail.ru (O.Y.F.); yelsufyevev@gmail.com (E.V.E.); karacharov@icct.ru (A.A.K.); 3 Laboratory of Quantum and Statistical Physics (LR18ES18), Faculty of Sciences, University of Monastir, Monastir 5079, Tunisia; issaoui_noureddine@yahoo.fr

**Keywords:** polysaccharides, sulfation, xanthan, structure, xanthan sulfate

## Abstract

Xanthan is an important polysaccharide with many beneficial properties. Sulfated xanthan derivatives have anticoagulant and antithrombotic activity. This work proposes a new method for the synthesis of xanthan sulfates using sulfamic acid. Various N-substituted ureas have been investigated as process activators. It was found that urea has the greatest activating ability. BBD of xanthan sulfation process with sulfamic acid in 1,4-dioxane has been carried out. It was shown that the optimal conditions for the sulfation of xanthan (13.1 wt% sulfur content) are: the amount of sulfating complex per 1 g of xanthan is 3.5 mmol, temperature 90 °C, duration 2.3 h. Sulfated xanthan with the maximum sulfur content was analyzed by physicochemical methods. Thus, in the FTIR spectrum of xanthan sulfate, in comparison with the initial xanthanum, absorption bands appear at 1247 cm^−1^, which corresponds to the vibrations of the sulfate group. It was shown by GPC chromatography that the starting xanthan gum has a bimodal molecular weight distribution of particles, including a high molecular weight fraction with M_w_ > 1000 kDa and an LMW fraction with M_w_ < 600 kDa. It was found that the Mw of sulfated xanthan gum has a lower value (~612 kDa) in comparison with the original xanthan gum, and a narrower molecular weight distribution and is characterized by lower PD values. It was shown by thermal analysis that the main decomposition of xanthan sulfate, in contrast to the initial xanthan, occurs in two stages. The DTG curve has two pronounced peaks, with maxima at 226 and 286 °C.

## 1. Introduction

Polysaccharides isolated from plant, animal, and bacterial raw materials have biocompatible, non-toxic, and biodegradable properties. Due to these qualities, they are actively used in pharmaceutical, biomedical, food, and cosmetic purposes [1].

Xanthan gum (XG) is a natural extracellular heteropolysaccharide produced by fermentation from *Xanthomonas campestris* [2]. The structure of XG contains a glucose molecule (β-1,4 glycosidic units) and also has acetate and pyruvate groups on the inner and terminal parts of the side chain, respectively [3,4,5]. Modification of XG with various functional groups opens up new possibilities for its use.

Among the well-known xanthan derivatives we distinguish: O-carboxymethyl XG [6], cationic [7,8] and amphoteric XG [9], hexadecyl XG [10], deacetylated XG [11], octyl XG [12], butyl XG [13], succinoyl XG [14], various forms of oxidized XG [2,15,16,17], and others. Modification of xanthan leads to the production of derivatives with specified characteristics of hydrophilic-hydrophobic properties for use in various fields.

It should be noted that among the various methods for the modification of xanthan, only one presents the sulfation method. Rafigh et al., 2020, carried out sulfation of xanthan with the dimethylformamide—sulfur trioxide (DMF-SO_3_) complex—and also carried out a detailed physicochemical study of the resulting product. The anticoagulant and antithrombotic activity of highly substituted xanthan sulfates have been shown [18].

Despite the obvious novelty in the preparation of xanthanum derivatives, the method [18] used the aggressive sulfating agent DMF-SO_3_ complex, obtained by adding chlorosulfonic acid to dimethylformamide. Currently, there is an alternative to the traditional method for the preparation of polysaccharide sulfates, based on the use of sulfamic acid in the presence of organic bases, both in the presence of organic solvents [19,20] and without them [21,22,23].

In this work, the xanthan sulfation process was optimized, the process activators based on urea were investigated, the initial and sulfated xanthan gum was analyzed by FTIR spectroscopy, XRD, AFM, thermal analysis, and GPC.

## 2. Materials and Methods

All chemicals were purchased from commercial suppliers. We used xanthan manufactured by Sigma-Aldrich (St. Louis, MO, USA). Sulfamic acid, 1,4-dioxane, and urea (Khimreaktivsnab, Republic of Bashkortostan, Ufa, Russia) were used in this work. Ethyl urea (Alfa Aesar, ThermoFisher GmbH, Kandel, Germany), methyl urea (J&K Scientific GmbH, Pudong District, Shanghai, China), and hydroxyethyl urea (Flourochem Ltd., Derbyshire, UK) were also used in this work.

### 2.1. Sulfation of Xanthan

A three-necked flask (100 mL) equipped with a thermometer, a glycerol bath, and a mechanical stirrer was used for the xanthan sulfation process, according to a modified procedure [19]. Xanthan (2.0 g), sulfamic acid, 1,4-dioxane (50 mL), and activator (urea, methyl urea, ethyl urea, hydroxyethyl urea) was stirred at 80–90 °C for 0.5–3.0 h (in accordance with the sulfation conditions given in Table 1). After the sulfation, the reaction mixture was cooled to room temperature and neutralized with 25% ammonia solution.

An MF-5030-46 MFPI dialysis bag (USA) (with a pore size of 3.5 kDa) was used for the purification (by dialysis) of a sulfated xanthan ammonium salt.

### 2.2. Statistical Analysis of the Sulfation Xanthan Process

Statgraphics Centurion XVI, DOE block (Design of Experiment) was used for the BBD of the xanthan sulfation process [24,25].

Three factors were included in the study as independent variables (their levels of variation are in parentheses): X_1_ is the amount of sulfating complex taken per 1 g of xanthan (1.5, 2.5, 3.5 mmol); X_2_ is the temperature of the xanthan sulfation process (70, 80, 90 °C), and X_3_ is the duration (0.5, 1.75, 3 h). The result of the sulfation process was characterized by the output parameter: Y_1_—sulfur content in xanthan sulfate (wt%). The choice of factors and ranges of their variation is based on [26,27,28,29,30,31].

The Box–Behnken experimental design (BBD) was used. The designations of the variables and the levels of their variation are shown in Table 2.

When carrying out the ANOVA analysis, we adopted a 95% level of significance (factors were determined by *p* < 0.05 values).

### 2.3. Methods of Physico-Chemical Analysis

#### 2.3.1. Elemental Analysis

For sulfated xanthan, elemental analysis was used via a FlashEA-1112 elemental analyzer (ThermoQuest, Waltham, Italy).

#### 2.3.2. FTIR

A Shimadzu IR Tracer-100 spectrometer (Shimadzu Corporation, Kyoto, Japan) was used for obtaining the FTIR spectra of initial xanthan and sulfated xanthan within the wavelength range of 400–4000 cm^−1^, as in [32].

#### 2.3.3. XRD

A DRON-3 X-ray diffractometer (CuKα monochromatized radiation (λ = 0.154 nm), voltage 30 kV, current 25 mA) was used for the X-ray diffraction phase analysis, as in [32].

#### 2.3.4. Gel Permeation Chromatography

An Agilent 1260 Infinity II Multi-Detector GPC/SEC System chromatograph was used for obtaining data on the average molecular mass (M_n_), average molecular weight (M_w_), and polydispersity of the initial and sulfated xanthan. For the separation, three PL aquagel-OH columns were used. The Agilent GPC/SEC MDS software was used for data analysis, as in [33].

#### 2.3.5. Atomic Force Microscopy

The obtained sulfated xanthan films were separated from the Petri dish with tweezers and analyzed by atomic force microscopy. The semi-contact AFM study of the sulfated xanthan films was carried out on an NT-MDT Solver P47 multimode scanning probe microscope (Moscow). Scanning was performed at no less than 3–4 points in several sites, as in [33]. The scanning rate was 1.5–2.0 Hz, and the image resolution was 256 × 256 pixels.

#### 2.3.6. Thermogravimetric Analysis

A NETZSCH STA 449 F1 Jupiter simultaneous thermal analysis instrument (Germany) was used for the thermogravimetric study, as in [23]. The thermal degradation of the samples was analyzed in argon in the temperature range from 30 to 600 °C; the protective and purge gas flow rates were 20 and 50 mL/min, respectively. The measurement results were processed using the NETZSCH Proteus—a Thermal Analysis.5.1.0 software that was supplied with the instrument.

## 3. Results and Discussion

### 3.1. The Role of the Activator in the Process of Sulfation of Xanthan with Sulfamic Acid

Nitrogen and sulfonated compounds have many beneficial properties [34,35,36,37].

**Figure 1:**
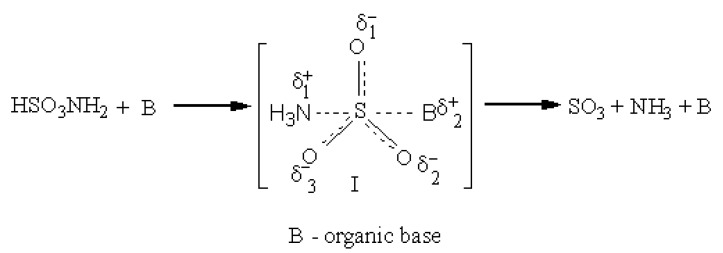
Scheme of the formation of a donor–acceptor complex of sulfamic acid with an organic base.

The mechanism of sulfation with sulfamic acid has not been previously studied in detail. There are suggestions [22,38,39,40,41] that when activators based on organic bases are used, a donor–acceptor complex is obtained, which is more capable of sulfating than the sulfamic acid (Figure 1). The limiting stage of sulfation is the conversion of an acid molecule with its decomposition to sulfur sodium oxide and ammonia [38,39,42]. Organic bases activate the process of sulfation of hydroxyl groups. This is due to the fact that the S–N bond in sulfamic acid is stronger than in the donor–acceptor complex [38,39,42].

In studies [38,39,43,44], some activators of this process are given: 1,4-dioxane, Urea, *N*,*N*-dimethylformamide, Morpholine, Piperidine, and Pyridine. In our work, a study of urea-based activators in the process of sulfation with sulfamic acid for the sulfur content in xanthan sulfates was performed. 1,4-dioxane was chosen as a solvent, as it showed its highest efficiency in the sulfation process with sulfamic acid [43,44].

According to the data shown in Table 1, in the absence of a catalyst, the process of sulfation of xanthan with sulfamic acid proceeds to a lesser extent than in the presence of activators. Among the studied activators, the lowest sulfur content in xanthan sulfate is achieved when using hydroxyethyl urea.

When ethyl urea is used, a product is obtained with a sulfur content of 0.6 wt%, more than when using hydroxyethyl urea. This can be due to several reasons. First, the lower ability of hydroxyethylurea to form a donor–acceptor complex with sulfamic acid, which in turn may be related to the basicity of hydroxyethylurea. Secondly, competing reactions of sulfation of the hydroxyl group of hydroxyethylurea are possible, which can reduce the content of sulfur trioxide in the reaction mass.

When methyl urea is used as an activator of the process of sulfation of xanthan with sulfamic acid, the product has a sulfur content of 8.7 wt%.

Thus, the activating ability decreases in the series: urea > methyl urea > ethyl urea > hydroxyethyl urea. That is, with an increase in the chain of a substituent in urea derivatives, their reactivity in sulfation reactions with sulfamic acid decreases. The reactivity of urea and its derivatives can also be associated with a change in the content of hydrogen bonds [45,46,47].

### 3.2. BBD Analysis of Xanthan Sulfation

We studied the effect of the sulfating complex amount, temperature, and duration of the process on the sulfur content in xanthan sulfates.

The experimental results are present in Table 3.

An increase in the temperature of the sulfation process should lead to an increase in the rate of both the addition of sulfate groups and the depolymerization of the polysaccharide macromolecule, but to a different extent. It is obvious that low molecular weight fractions of xanthan, which also exhibit high reactivity in the sulfation reaction, are the most prone to depolymerization (hydrolysis). With an increase in the temperature of the sulfation process, the amount of low molecular weight product with a high sulfur content begins to increase over time, which is removed during dialysis cleaning (see “Section 3.5 Gel Permeation Chromatography”).

According to Table 3, the highest sulfur content in xanthan sulfate is achieved at a process temperature of 90 °C, duration of 1.75 h, and an amount of sulfating complex of 3.5 mmol. A further increase in both the duration and temperature can lead to hydrolysis reactions and partial destruction of xanthan molecules under the action of sulfamic acid. It should be noted that a smaller amount of the sulfating complex leads to lower values of the sulfur content in xanthan sulfate, which can probably be associated with more regularly occurring hydrolysis processes [26].

The results of analysis of variance are given in Table 4.

The Box–Behnken optimization has been found to be useful for developing an accurate experimental model among the significant factors [48]. Analysis of variance (ANOVA) was used to analyze data on the sulfur content of xanthan sulfates obtained in the experiment (Table 3). Significant factors were determined by *p* < 0.05 values. For all independent variables this factor was <0.0007.

The result showed the regression model used to study the effect of the explanatory variables on the sulfur content of xanthan sulfates was accurate. This is also indicated by high values of F > 55 (Table 4). According to Table 4, all explanatory variables contribute to the overall output parameter variance.

The resulting regression equation (second order polynomial) (Equation (1)), which explains the normal logarithm of the response as the mean of three factors (independent) and their functions, despite their significance.

Y_1_ = −8.76129 + 1.18333X_1_ − 0.0083333X_2_ + 7.28933X_3_ − 0.404167X_1_^2^ + 0.04X_1_X_2_ − 0.3X_1_X_3_ + 0.00070833X_2_^2^ − 0.02X_2_X_3_ − 0.962667X_3_^2^ (1)

**Figure 2:**
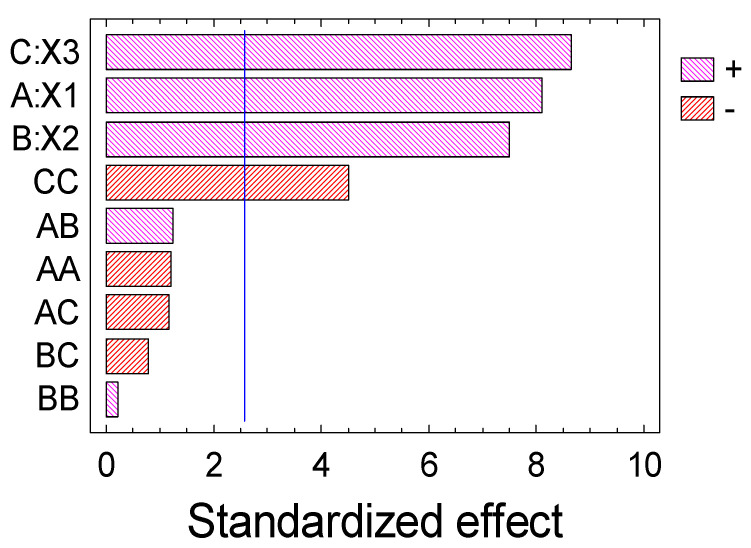
Pareto graph of significant variables.

ANOVA (Table 4) and a Pareto graph (Figure 2) for the three factors explain that the Box–Behnken quadratic model can be sufficiently applied to simulate the xanthan (Y) sulfation process.

**Figure 3:**
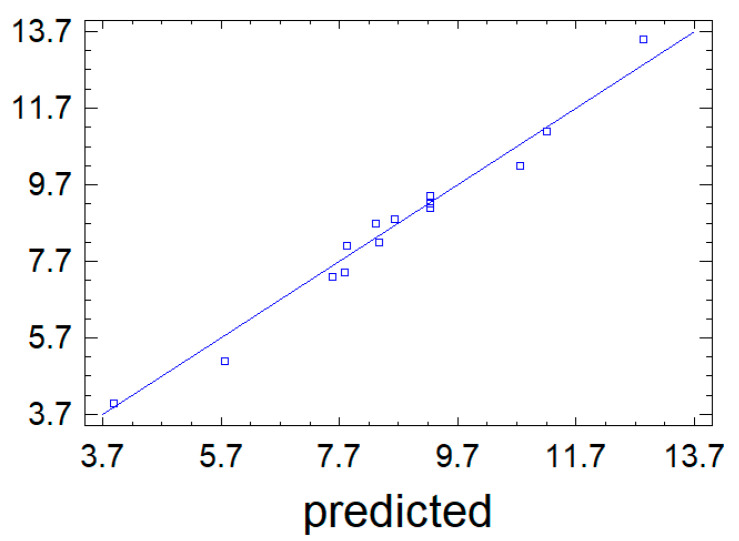
The results of observations against the values of the output parameter Y_1_ predicted by the mathematical model (1).

According to Equation (1), the mathematical model is accurate since the points in Figure 3 lie closer to the straight line, which also shows good predictive properties of the equation.

**Figure 4:**
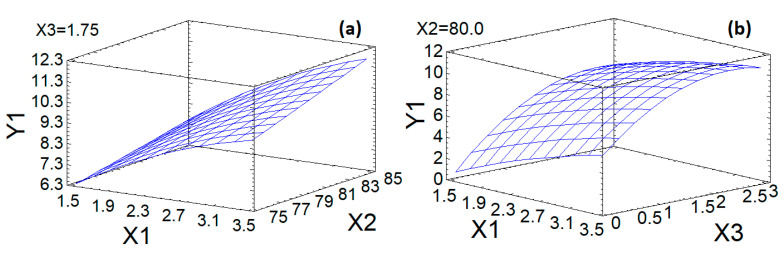

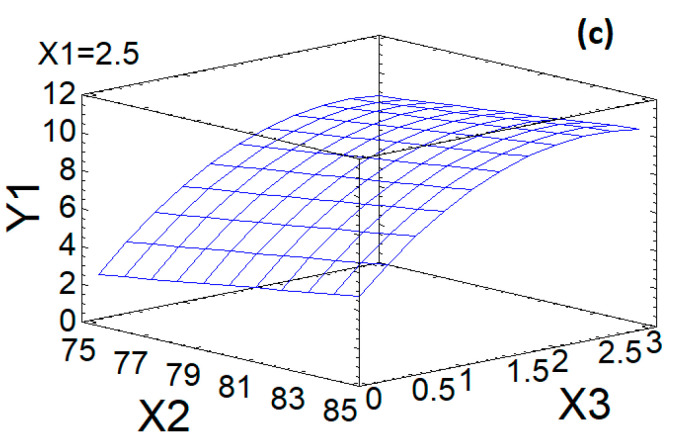
Response surface of output parameters with different effects of experimental conditions: (**a**)—Influence of factors X_1_ and X_2_ on Y_1_; (**b**)—Influence of factors X_1_ and X_3_ on Y_1_; (**c**)—Influence of factors X_2_ and X_3_ on Y_1_.

A graphical display of Equation (1) in the form of a response surface is shown in Figure 4.

The dependence of the sulfur content on variable factors—the amount of the sulfating complex and the temperature of the xanthan sulfation process—in the form of a response surface has an almost flat appearance without significant bends (Figure 4a). For this dependence, a maximum is observed at the maximum values of the factors X_1_ and X_2_ within the accepted experimental conditions.

The response surface, reflecting the dependence of the output parameter—the sulfur content on the variable factors—the amount of the sulfating complex, and the duration of the sulfation process, has the form of a curved plane with maxima for X_1_ and X_3_ of 3.5 and 2.3, respectively (Figure 4b).

The dependence of the output parameter (sulfur content in xanthan sulfate) on variable factors of the temperature and duration of the xanthan sulfation process in the form of a response surface has the form of a curved plane that reaches a plateau after X_3_ values of 2.3 h (Figure 4c).

The coefficient of determination is *R*^2^_adj_ = 93.8%. This testifies to the adequacy of Equation (1) to the observation results and allows using it as a mathematical model of the process under study.

The calculated optimal conditions for the sulfation of xanthan with sulfamic acid in 1,4-dioxane in the presence of urea (to obtain xanthan sulfate with a sulfur content of 13.1 wt%) are: the amount of sulfating complex per 1 g of xanthan 3.5 mmol, temperature 90 °C, and duration 2.3 h.

### 3.3. FTIR Spectroscopy

**Figure 5:**
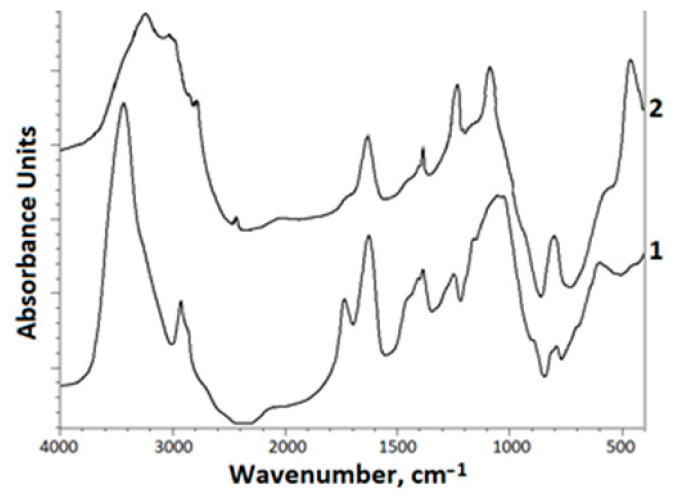
FTIR-spectra: 1—initial xanthan, 2—sulfated xanthan.

The characterization of the parent and sulfated xanthan was performed by FTIR analysis (Figure 5). The FTIR spectra of the starting xanthan gum contain functional groups of carbonyl, carboxyl, and acetal groups in xanthan gum [5,49].

The main FTIR peaks remained almost unchanged after sulfate modification. The introduction of a sulfate group into a xanthan molecule changes the FTIR spectra. Thus, absorption bands appear at 1247 cm^−1^, which correspond to the vibrations of the sulfate group. In the FTIR spectrum of sulfated xanthan, in comparison with the initial xanthan, there is no absorption band at 1735 cm^−1^, and there is a noticeable decrease in the band at 1624 cm^−1^, corresponding to the vibrations of the ionized carboxyl group. In addition, in sulfated xanthan gum, there is an absorption band at ~810 cm^−1^, which, in comparison with the band at 801 cm^−1^, is present in the FTIR spectrum of the starting xanthan gum, has a high intensity. This change in the nature of the FTIR spectrum confirms the introduction of the sulfate group into the xanthan molecule. The peak at 810 cm^−1^ is typical. In addition, the absorption peak at 810 cm ^−1^ is typical of C–O–S stretching. This absorption band is associated with bending vibrations of C–O–S bonds [50].

The peak at 1026 cm^−1^ is due to the stretching vibration of the C–O alcohol groups [51,52,53]. The absorption band at 2924 cm^−1^ corresponds to the vibrations of the CH_2_ group.

The introduction of a sulfate group into a xanthan molecule changes the FTIR spectra. Thus, absorption bands appear at 1247 cm^−1^, which correspond to the vibrations of the sulfate group. In the FTIR spectrum of sulfated xanthan, in comparison with the initial xanthan, there is no absorption band at 1735 cm^−1^, and there is a noticeable decrease in the band at 1624 cm^−1^, corresponding to the vibrations of the carbonyl group.

A decrease in the intensity of these absorption bands can also be associated with the partial hydrolysis of the side chains of xanthan gum during the synthesis (see “Section 3.5. Gel Permeation Chromatography”).

### 3.4. X-ray Diffractions Analysis

**Figure 6:**
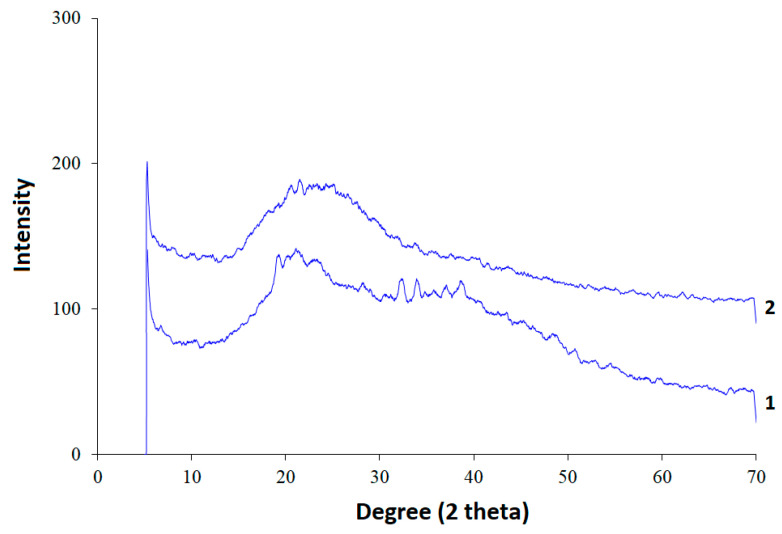
XRD data: 1—initial xanthan, 2—sulfated xanthan.

The amorphous structures observed in the xanthan sulfate sample were probably the result of a structural contribution from the parent xanthan as well (Figure 6). The amorphous properties of xanthan were confirmed by a broad diffraction peak at 2θ = 22.1°, probably as a result of its double helix conformation [16,54]. In the process of sulfation, an increase in the amorphization of the initial xanthan structure was observed, which was manifested by a decrease in the intensity in X-ray diffraction patterns from 17° to 50° 2θ.

It is known [23,43,55,56,57,58,59] that sulfation of polysaccharides leads to greater amorphization of their structure. Thus, the data shown in Figure 6 are in good agreement with the literature.

Polysaccharides with an amorphous structure (Figure 6) are more susceptible to modification (including hydrolysis) of glycosidic bonds under the action of acids [23].

### 3.5. Gel Permeation Chromatography

**Figure 7:**
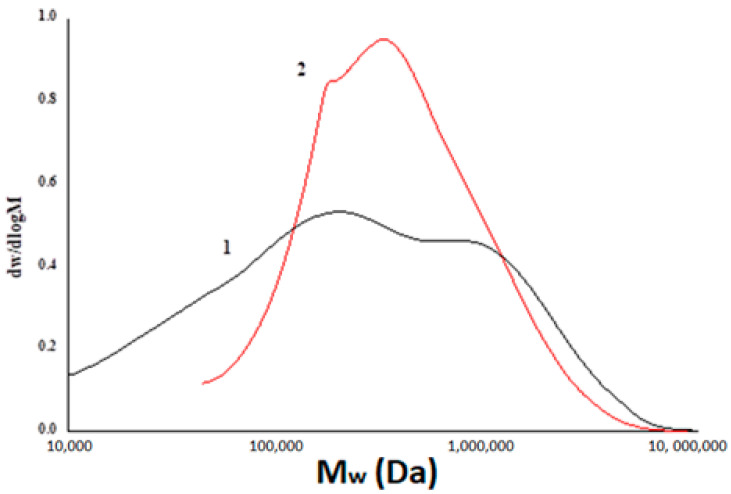
Molecular weight distribution of xanthan (1) and sulfated xanthan (2) samples.

GPC data of sulfated xanthan shows the decreasing of the sulfated xanthan molecular weight compared to the original xanthan (Figure 7, Table 5). It is known [23] that glycosidic bonds in hemicelluloses are destroyed by the action of acids. In our case, under the action of sulfamic acid, in addition to the addition of the sulfate group, the hydrolysis reaction is also observed (Figure 7); therefore, the treatment of xanthan with acids causes its depolymerization.

The initial xanthan sample has a molecular weight (M_w_) of ~620 kDa and a fairly high PD value (7.44), which indicates the presence of a large number of branches containing acetate and pyruvate groups on the inner and terminal parts of the side chain, respectively [18]. It was found that the original xanthan gum has a bimodal molecular weight distribution of particles (Figure 7), which includes a high molecular weight fraction with M_w_ > 1000 kDa and a low molecular weight fraction with M_w_ < 600 kDa. After the sulfation process, a redistribution of molecular weights in the sample was observed (Figure 7). It was found that the Mw of sulfated xanthan gum has a lower value (~612 kDa) in comparison with the initial xanthan gum and a narrower molecular weight distribution and is characterized by lower PD values (2.34). At the same time, the proportion of the high-molecular-weight fraction in the sample decreases noticeably, and the peak corresponding to the low-molecular-weight fraction becomes less pronounced. This can be explained by the fact that sulfation of xanthan with sulfamic acid in combination with 1,4-dioxane and urea can lead to the partial destruction of the XG structure, partial hydrolysis of the polymer chain in an acidic medium, and the elimination of side branches.

### 3.6. Thermal Analysis

**Figure 8:**
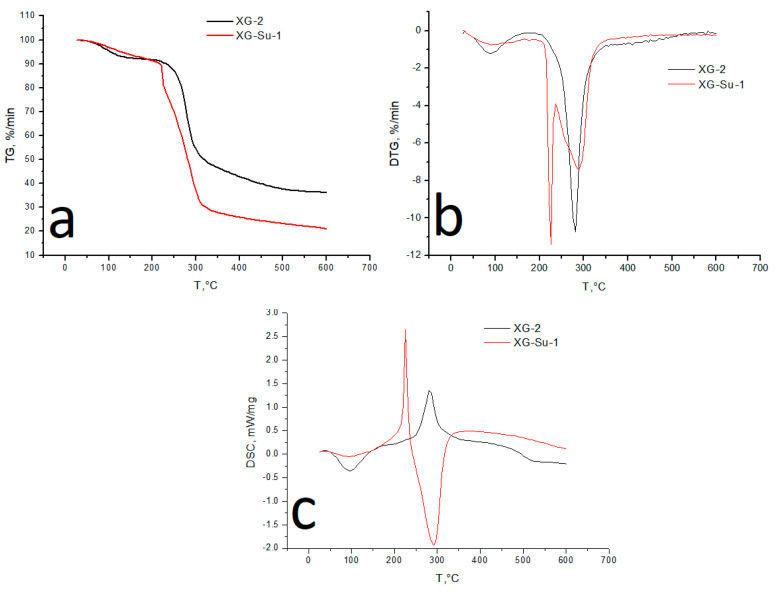
TG (**a**), DTG (**b**), and DSC (**c**) curves of xanthan and xanthan sulfate.

Analysis of the TG and DTG curves (Figure 8a,b) demonstrates a decrease in the mass of the initial xanthanum (−8.1%) in the range from 30 to 180 °C, which is probably due to the desorption of moisture from the sample surface and from the bulk, as a result of the rupture of hydrogen bonds between water molecules and polar functional groups. On the DSC curve (Figure 8c), this process corresponds to a distinct endothermic peak. The rate of weight loss for xanthan increases with increasing temperature up to 280.74 °C and then decreases. Thus, the main degradation of xanthan occurs in one step. In the range of the main decomposition (up to 300 °C), xanthan “loses” 46% of the initial sample, and by 600 °C, the weight loss was 64%. On the DSC curve, the main decomposition of the xanthan structure is characterized by a well-discernible exothermic effect with a maximum at 281 °C.

For sulfated xanthan, the initial stage of heating (up to 180 °C) is characterized by a less intense weight loss (−7.7%), compared to the original xanthan. The endothermic effect on the DSC curve of the indicated interval is flatter and not as pronounced.

The main decomposition of xanthan sulfate, in contrast to the original xanthan, occurs in two stages. The DTG curve has two pronounced peaks, with maxima at 226 and 286 °C. The first stage is probably associated with the decomposition of sulfo groups (weight loss 18.8%). During the second stage of decomposition (up to 300 °C), xanthan “loses” 64%. On the DSC curve, the decomposition of sulfo groups corresponds to a sharp and rather intense exothermic peak, with a maximum at 226 °C, which, with an increase in the heating temperature, passes into the endothermic zone. The endothermic effect in this region is probably associated with the melting of the substance with the simultaneous decomposition of the structure of xanthan sulfate; the latter statement is confirmed by the slope of the TG curve in this interval. Further heating of the sample to 600 °C leads to aromatization of the structure with the formation of a carbonized residue (weight loss 79%).

### 3.7. Atomic Force Microscopy

**Figure 9:**
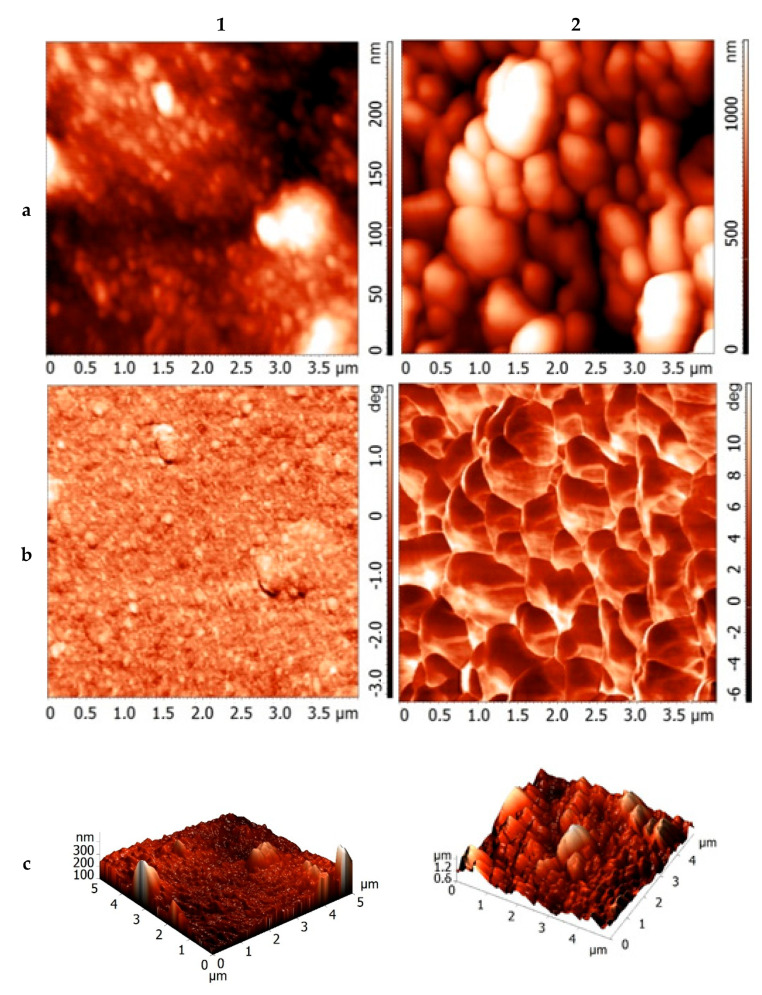

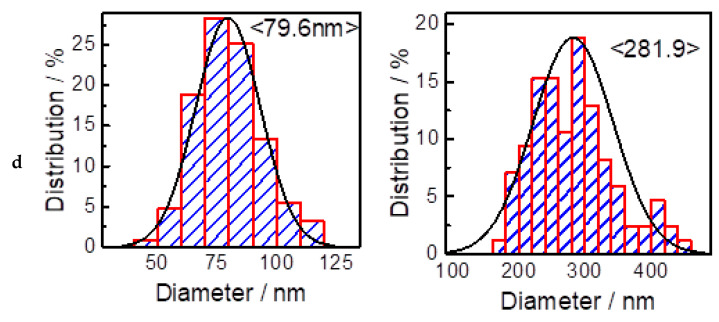
AFM data on (1) initial xanthan and (2) xanthan sulfate (relief (**a**), phase contrast (**b**), 3D relief (**c**), and particle size distribution (**d**)).

According to Figure 9, the surface of the xanthan film consists of near-spherical particles with an average size of 79.6 nm. Sulfation of xanthan leads to an increase in spherical particles to an average size of 281.9 nm as a result of agglomeration and aggregation. It was previously reported that the aggregation of a polysaccharide can be increased by introducing functional groups (for example, sulfate groups) that increase its inter and intramolecular interactions or polyelectrolyte effects [60,61,62]. Thus, the results obtained in our work correspond to the literary sources.

According to the phase-contrast data (Figure 9b), the surface of the films of the initial and sulfated xanthan gum does not contain impurities.

The particle size distribution (Figure 9d) has the form of a normal distribution (Gaussian distribution), both for the original and for sulfated xanthan.

The embedding of a sulfate group into a xanthan molecule decreases its molecular weight and also leads to an increase in amorphism and aggregation.

## 4. Conclusions

In this work, we proposed a new method for the synthesis of xanthan polysaccharides using sulfamic acid upon activation with urea derivatives. It has been shown that urea has the highest activating activity in the reaction of sulfation of xanthan with sulfamic acid. The influence of the amount of the sulfating complex, the temperature, and the duration of the sulfation process on the sulfur content in xanthan sulfate has been established. It has been shown that the optimal conditions for xanthan sulfation are: the amount of sulfating complex per 1 g of xanthan is 3.5 mmol, the temperature is 90 °C, and the duration is 2.3 h. The reaction product with the maximum sulfur content was analyzed by FTIR spectroscopy, X-ray diffraction, gel penetration microscopy, thermal analysis, and atomic force microscopy. According to the data of gel permeation chromatography, a decrease in molecular weight is observed as a result of sulfation, which also indicates side hydrolysis reactions. According to thermal analysis, sulfated xanthan is less thermostable than the parent xanthan; however, the main weight loss for it occurs at temperatures above 226 °C, which makes it acceptable for use as a biologically active substance.

In the future, xanthan sulfates can be used as an anticoagulant and hypolipidemic substance [63], a polyanion [64], as well as for the preparation of interpolyelectrolyte complexes [65].

## Figures and Tables

**Table 1:** Influence of the activator of the sulfation reaction with sulfamic acid on the sulfur content in xanthan sulfate (temperature 90 °C, time 3 h, the amount of sulfating complex 3.5 mmol per 1 g of xanthan).

No.	Activator	Formula	Sulfur Content % wt.
1	Without activator	-	3.2
2	Urea	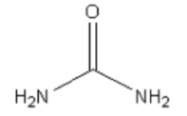	13.5
3	Methyl urea	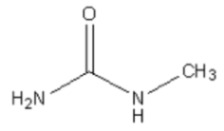	8.7
4	Ethyl urea	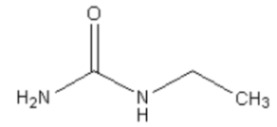	7.9
5	Hydroxyethyl urea	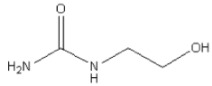	7.3

**Table 2:** Independent factors and output parameters (experimental results).

Factors and Parameters	Notation in Equations	Range of Variation
Amount of sulfating complex, mmol	X_1_	1.5–3.5
Temperature, °C	X_2_	75–85
Duration of process, h	X_3_	0.5–3.0
Sulfur content, wt%	Y_1_	-

**Table 3:** Influence of xanthan sulfation conditions with SAA in 1,4-dioxane with urea on sulfur content in xanthan sulfates.

No.	Sulfating Complex Amount, mmol	Temperature, °C	Duration of Process, h	Sulfur Content, wt%
1	2.5	80	1.75	9.1
2	1.5	70	1.75	5.1
3	3.5	70	1.75	8.8
4	1.5	90	1.75	8.2
5	3.5	90	1.75	13.5
6	1.5	80	0.5	3.7
7	3.5	80	0.5	7.3
8	2.5	80	1.75	9.4
9	1.5	80	3	8.1
10	3.5	80	3	10.2
11	2.5	70	0.5	4.0
12	2.5	90	0.5	7.4
13	2.5	70	3	8.7
14	2.5	90	3	11.1
15	2.5	80	1.75	9.2

**Table 4:** The result of the analysis of variance.

Sources of Variance	Statistical Characteristics	
	*F*-Ratio	*p*-Value
X_1_ X_2_ X_3_ X_1_^2^ X_1_X_2_ X_1_X_3_ X_2_^2^ X_2_X_3_ X_3_^3^	65.59 56.14 74.81 1.46 1.55 1.37 0.04 0.61 20.28	0.0005 0.0007 0.0003 0.2803 0.2678 0.2952 0.8404 0.4712 0.0064
*Df* *R* ^2^ *R* ^2^_adj_	14 97.8 93.8	

**Table 5:** Number average molecular weight (M_n_), weight average molecular weight (M_w_), and polydispersity (PD) of xanthan gum samples and its sulfated derivative.

Samples	M_n_, Da	M_w_, Da	PD
Xanthan	83,412	620,439	7.44
Sulfated xanthan	261,497	611,935	2.34

## Data Availability

All data generated during this study are included in this article.
